# Draft genome sequences from 127 *Legionella* spp. strains isolated in water systems linked to legionellosis outbreaks

**DOI:** 10.1128/mra.01154-23

**Published:** 2024-05-01

**Authors:** Andrea Colautti, Marcello Civilini, Marinella Franchi, Antonella Felice, Stefano De Martin, Emanuele De Paoli, Michele Vidotto, Lucilla Iacumin

**Affiliations:** 1 Department of Agricultural, Food, Environmental and Animal Science (Di4A), University of Udine, Udine, Italy; 2 Laboratory of Microbiology, ARPA–Regional Agency for Environmental Protection Friuli Venezia Giulia, Udine, Italy; 3 IGA Technology Services S.r.l., Udine, Italy; University of Maryland School of Medicine, Baltimore, Maryland, USA

**Keywords:** *Legionella pneumophila*, genome, WGA-LP, serogroup 1, legionellosis, legionnaire's disease

## Abstract

Legionnaires' disease is a severe form of pneumonia caused by *Legionella* spp. bacteria. According to the European Centre for Disease Prevention and Control, problems related to this pathogen showed a significant surge in recent years, making its monitoring critical.

## ANNOUNCEMENT

According to the latest report from the European Centre for Disease Prevention and Control (ECDC), Europe suffered the highest annual Legionnaires' disease notification rate to date (1), and climate change may play a role in its spread in the coming years (2). However, cases are rarely culture-confirmed, potentially leading to misinterpretations of pathogen diffusion dynamics and underestimations of Legionnaires' disease cases caused by *Legionella* species other than *Legionella pneumophila* (1).

In light of these considerations, to further our understanding of the spread of this pathogen in the Italian region of Friuli Venezia Giulia, 127 *Legionella* spp. strains were sequenced, both to confirm the species and to analyze their genetic traits. These strains were isolated as part of national surveillance plans conducted between 2005 and 2017 by the Regional Agency for the Protection of the Environment of Friuli Venezia Giulia (ARPA FVG), from tap water of different types of buildings identified as sources of legionellosis outbreaks, with sampling and analysis protocols carried out in accordance with Italian (3) and international directives (4).

Isolates were cultured on Buffered Charcoal Yeast Extract Agar (BCYEA) supplemented with Legionella BCYE Growth Supplement with and without cysteine (Biolife, Italy). Presumptive positive colonies were further confirmed by qualitative real-time PCR using the IQ-Check screen *L. pneumophila* Kit (Bio-Rad, Italy) and serologically identified using *Legionella* rapid latex test Kit (Biolife, Italy) monovalent antisera (5).

For DNA extraction, cultures cryopreserved at −80°C in Microbanks (Pro Lab Diagnostics, Canada) were revitalized by streaking onto BCYEA and cultivated under microaerophilic conditions at 36°C for 5 days. The DNA was then extracted using the MagAttract HMW DNA kit (Qiagen, Germany) and purified using the Genomic DNA Clean & Concentrator kit (Zymo Research, USA). The extracted DNA was checked for purity and quantified both via NanoDrop (Thermo Scientific, USA) and Qubit 3 Fluorometer (Invitrogen, USA) using the Qubit dsDNA BR Assay kit (Invitrogen, USA). The quality was then analyzed by agarose gel electrophoresis (0.8% agarose, 1× TAE buffer). Libraries were prepared sonicating the DNA with a BioRuptor sonicator (Diagenode, USA), subsequently using the Celero DNA-Seq kit (Tecan, Switzerland). The size of the individual fragments composing the library was measured using BioAnalyzer 2100 DNA chip electrophoresis (Agilent Technologies, USA). Paired-end sequencing of the libraries was then performed on the Illumina MiSeq platform (Illumina, San Diego, USA).

Raw reads (300 bp length) were carefully processed and assembled through the WGA-LP pipeline (6) using the following included tools in default mode. The reads were qualitatively checked through FastQC v0.11.9 (7) and trimmed with Trimmomatic v0.39 (8), verifying the presence of contaminations through Kraken2 v2.0.8-b (9). For the assembling process, SPAdes v3.15.2 was used as assembler (10), evaluating the quality and completeness of the final assemblies using CheckM v1.1.3 (11), Quast v5.0.2 (12), and Merqury v1.3 (13). The assembled genomes were then annotated using PGAP 2022-04-14.build6021 (14). After ANI (15) and dDDH (16) evaluation, the strains were identified as 1 *L*. *taurinensis*, 117 *L*. *pneumophila*, and 9 *L*. *pneumophila* subsp. *fraseri* that were not distinguished by conventional techniques.

**TABLE 1:** Statistics of assembled genomes

Strain	Species	Isolation structure	SRA	Total reads	GCA	Total length (bp)^*a*^	Contigs	Coverage^*b*^	Genes (total)^*a*^	N50^*c*^	GC (%)^*c*^	Completeness (%)^*d*^	Presumptive Contamination^*d*^
LP1_2005_3688	*L. pneumophila*	Healthcare facility	SRR26709269	1,219,870	JAWVHS000000000	3,449,728	58	127	3,118	238,579	38.26	100	0.00
LP3_2010_7343	*L. pneumophila*	Nursing home	SRR26709268	714,743	JAWVHT000000000	3,495,876	120	68	3,180	83,435	38.38	100	0.00
LP4_2010_12257	*L. pneumophila*	Healthcare facility	SRR26709202	761,509	JAWVHU000000000	3,697,419	46	77	3,334	237,645	38.22	100	0.00
LP5_2010_12508	*L. pneumophila*	Nursing home	SRR26709191	623,974	JAWVHV000000000	3,558,565	108	65	3,228	77,780	38.31	100	0.00
LP6_2010_13100	*L. pneumophila*	Lodging facility	SRR26709295	583,028	JAWVHW000000000	3,592,706	108	60	3,265	81,328	38.28	100	0.00
LP7_2010_13335	*L. pneumophila*	Nursing home	SRR26709284	878,150	JAWVHX000000000	3,419,937	97	118	3,117	138,459	38.19	100	0.58
LP8_2010_13575	*L. pneumophila*	Industry	SRR26709273	450,681	JAWVHY000000000	3,546,940	58	60	3,227	177,103	38.29	100	0.00
LP9_2010_13639	*L. pneumophila*	Healthcare facility	SRR26709231	395,285	JAWVHZ000000000	3,372,946	21	42	3,036	395,932	38.25	100	0.34
LP10_2010_14262	*L. pneumophila*	Nursing home	SRR26709220	489,130	JAWVIA000000000	3,654,606	59	62	3,343	205,207	38.31	100	0.58
LP11_2010_14388	*L. pneumophila*	Nursing home	SRR26709181	603,291	JAWVIB000000000	3,374,138	50	83	3,042	193,144	38.21	100	0.58
LP12_2010_14946	*L. pneumophila*	Healthcare facility	SRR26709267	722,472	JAWVIC000000000	3,402,346	24	100	3,052	502,991	38.23	100	0.58
LP13_2010_15075	*L. pneumophila* subsp. *fraseri*	Lodging facility	SRR26709256	244,070	JAWVID000000000	3,402,493	83	23	3,098	82,206	38.16	99.71	0.00
LP14_2011_1360	*L. pneumophila*	Swimming pool	SRR26709245	297,781	JAWVIE000000000	3,592,880	121	27	3,276	79,633	38.27	100	0.00
LP15_2011_2680	*L. pneumophila* subsp. *fraseri*	Lodging facility	SRR26709209	489,711	JAWVIF000000000	3,402,542	74	69	3,096	90,333	38.16	99.71	0.00
LP16_2011_2730	*L. pneumophila*	Nursing home	SRR26709208	519,393	JAWVIG000000000	3,749,943	48	66	3,388	237,645	38.25	100	0.00
LP17_2011_4965	*L. pneumophila*	Healthcare facility	SRR26709207	1,517,793	JAWVIH000000000	3,375,118	29	202	3,046	430,669	38.27	100	0.00
LP18_2011_5016	*L. pneumophila*	Healthcare facility	SRR26709206	2,154,403	JAWVII000000000	3,395,310	51	284	3,076	405,459	38.21	100	0.00
LP19_2011_5017	*L. pneumophila*	Healthcare facility	SRR26709205	834,713	JAWVIJ000000000	3,650,565	133	81	3,321	78,758	38.27	100	0.00
LP20_2011_5054	*L. pneumophila*	Healthcare facility	SRR26709204	654,650	JAWVIK000000000	3,580,244	50	85	3,258	194,234	38.23	100	0.00
LP21_2011_6872	*L. pneumophila*	Healthcare facility	SRR26709203	1,120,333	JAWVIL000000000	3,491,586	55	138	3,143	178,388	38.31	100	0.00
LP22_2011_7143	*L. pneumophila*	Lodging facility	SRR26709201	507,859	JAWVIM000000000	3,595,000	145	44	3,292	77,756	38.28	100	0.00
LP23_2011_7324	*L. pneumophila*	Healthcare facility	SRR26709200	1,220,667	JAWVIN000000000	3,376,825	29	152	3,046	657,397	38.24	100	0.00
LP24_2011_7512	*L. pneumophila*	Private residence	SRR26709199	703,196	JAWVIO000000000	3,633,830	49	92	3,273	204,409	38.23	100	0.00
LP25_2011_7660	*L. pneumophila*	Lodging facility	SRR26709198	514,348	JAWVIP000000000	3,451,422	49	71	3,118	194,234	38.26	100	0.00
LP26_2011_7708	*L. pneumophila*	Healthcare facility	SRR26709197	670,876	JAWVIQ000000000	3,585,672	46	91	3,257	252,775	38.22	100	0.00
LP27_2011_7798	*L. pneumophila*	Swimming pool	SRR26709196	278,970	JAWVIR000000000	3,448,180	43	39	3,105	237,645	38.26	100	0.00
LP28_2011_9951	*L. pneumophila*	Nursing home	SRR26709195	1,358,309	JAWVIS000000000	3,350,221	29	169	3,020	657,620	38.24	100	0.58
LP30_2015_4925	*L. pneumophila*	Healthcare facility	SRR26709194	366,224	JAWVIT000000000	3,587,180	73	40	3,266	125,723	38.23	100	0.00
LP31_2015_4961	*L. pneumophila*	Healthcare facility	SRR26709193	447,121	JAWVIU000000000	3,585,729	62	57	3,259	168,307	38.22	100	0.00
LP32_2015_6923	*L. pneumophila*	Agritourism	SRR26709192	427,925	JAWVIV000000000	3,326,317	46	58	2,979	297,958	38.23	100	0.00
LP33_2015_6924	*L. pneumophila*	Agritourism	SRR26709190	415,889	JAWVIW000000000	3,333,309	52	51	2,988	196,896	38.23	100	0.00
LP34_2015_6925	*L. pneumophila*	Agritourism	SRR26709189	549,476	JAWVIX000000000	3,333,656	47	78	2,988	300,988	38.23	100	0.00
LP36_2015_9499	*L. pneumophila*	Private residence	SRR26709188	671,671	JAWVIY000000000	3,404,566	35	96	3,070	243,407	38.23	100	0.58
LP37_2015_9502	*L. pneumophila*	Private residence	SRR26709187	304,947	JAWVIZ000000000	3,406,006	48	37	3,073	143,323	38.23	100	0.58
LP38_2015_9504	*L. pneumophila*	Private residence	SRR26709186	903,173	JAWVJA000000000	3,405,331	30	125	3,064	243,278	38.24	100	0.58
LP39_2015_9507	*L. pneumophila*	Private residence	SRR26709185	273,327	JAWVJB000000000	3,404,180	34	31	3,073	223,866	38.23	100	0.58
LP40_2015_11509	*L. pneumophila*	Private residence	SRR26709184	510,489	JAWVJC000000000	3,461,502	53	42	3,151	178,815	38.28	100	0.00
LP41_2015_11525	*L. pneumophila*	Private residence	SRR26709183	919,133	JAWVJD000000000	3,462,581	48	119	3,146	259,182	38.28	100	0.00
LP42_2015_11526	*L. pneumophila*	Private residence	SRR26709172	265,161	JAWVJE000000000	3,460,919	48	30	3,146	187,646	38.28	100	0.00
LP43_2015_11527	*L. pneumophila*	Private residence	SRR26709296	903,019	JAWVJF000000000	3,467,071	44	114	3,147	259,182	38.28	100	0.00
LP44_2015_11755	*L. pneumophila*	Lodging facility	SRR26709294	1,124,039	JAWVJG000000000	3,466,156	42	160	3,141	257,884	38.28	100	0.00
LP45_2015_11756	*L. pneumophila*	Lodging facility	SRR26709293	740,211	JAWVJH000000000	3,373,607	54	106	3,047	248,989	38.21	100	0.00
LP46_2015_11762	*L. pneumophila*	Lodging facility	SRR26709292	273,569	JAWVJI000000000	3,463,769	45	30	3,143	236,421	38.28	100	0.00
LP47_2015_11763	*L. pneumophila*	Lodging facility	SRR26709291	355,201	JAWVJJ000000000	3,465,516	45	42	3,147	257,884	38.28	100	0.00
LP48_2015_11765	*L. pneumophila*	Lodging facility	SRR26709290	698,763	JAWVJK000000000	3,371,735	42	79	3,038	248,989	38.21	100	0.58
LP49_2015_12212	*L. pneumophila*	Private residence	SRR26709289	524,790	JAWVJL000000000	3,373,206	53	64	3,046	248,989	38.21	100	0.58
LP50_2015_12213	*L. pneumophila*	Private residence	SRR26709288	581,796	JAWVJM000000000	3,372,517	46	69	3,043	248,989	38.21	100	0.58
LP51_2015_12754	*L. pneumophila*	Healthcare facility	SRR26709287	871,158	JAWVJN000000000	3,586,496	59	107	3,260	168,307	38.23	100	0.00
LP52_2015_12755	*L. pneumophila*	Healthcare facility	SRR26709286	1,029,339	JAWVJO000000000	3,585,896	59	109	3,256	168,307	38.23	100	0.00
LP53_2015_12756	*L. pneumophila*	Healthcare facility	SRR26709285	434,524	JAWVJP000000000	3,584,499	50	51	3,252	168,307	38.23	100	0.00
LP54_2015_12757	*L. pneumophila* subsp. *fraseri*	Healthcare facility	SRR26709283	662,187	JAWVJQ000000000	3,450,390	59	98	3,147	202,165	38.19	100	0.00
LP56_2015_13149	*L. pneumophila* subsp. *fraseri*	Agritourism	SRR26709282	556,590	JAWVJR000000000	3,448,577	53	73	3,140	194,988	38.19	100	0.00
LP57_2015_13150	*L. pneumophila* subsp. *fraseri*	Agritourism	SRR26709281	490,106	JAWVJS000000000	3,449,322	54	66	3,153	233,885	38.19	100	0.00
LP58_2015_13151	*L. pneumophila* subsp. *fraseri*	Agritourism	SRR26709280	580,581	JAWVJT000000000	3,449,725	58	85	3,145	233,885	38.19	100	0.00
LP59_2015_13152	*L. pneumophila*	Agritourism	SRR26709279	610,972	JAWVJU000000000	3,467,694	43	76	3,146	257,885	38.29	100	0.00
LP60_2015_13153	*L. pneumophila* subsp. *fraseri*	Agritourism	SRR26709278	588,253	JAWVJV000000000	3,449,539	54	85	3,147	194,987	38.19	100	0.00
LP61_2015_13154	*L. pneumophila*	Agritourism	SRR26709277	619,082	JAWVJW000000000	3,462,904	31	80	3,132	257,884	38.28	100	0.00
LP62_2015_13155	*L. pneumophila*	Agritourism	SRR26709276	1,471,905	JAWVJX000000000	3,463,554	30	170	3,135	257,884	38.29	100	0.00
LP63_2016_1544	*L. pneumophila*	Commercial laundry	SRR26709275	595,548	JAWVJY000000000	3,373,751	44	66	3,048	221,653	38.27	100	0.00
LP64_2016_1756	*L. pneumophila*	Healthcare facility	SRR26709274	304,853	JAWVJZ000000000	3,411,223	21	43	3,077	469,611	38.26	100	0.00
LP65_2016_1763	*L. pneumophila*	Healthcare facility	SRR26709272	430,977	JAWVKA000000000	3,395,170	38	37	3,070	319,785	38.23	100	0.00
LP66_2016_1764	*L. pneumophila*	Healthcare facility	SRR26709271	336,418	JAWVKB000000000	3,396,681	25	51	3,063	469,649	38.23	100	0.00
LP67_2016_1798	*L. pneumophila*	Healthcare facility	SRR26709270	513,475	JAWVKC000000000	3,391,583	55	55	3,052	155,505	38.24	100	0.58
LP68_2016_1801	*L. pneumophila*	Healthcare facility	SRR26709238	312,585	JAWVKD000000000	3,518,580	72	46	3,190	126,632	38.27	100	0.00
LP69_2016_2458	*L. pneumophila*	Private residence	SRR26709237	384,399	JAWVKE000000000	3,480,717	59	57	3,173	213,317	38.26	100	0.00
LP70_2016_2460	*L. pneumophila*	Private residence	SRR26709236	339,623	JAWVKF000000000	3,481,141	74	47	3,180	166,059	38.27	100	0.00
LP71_2016_4320	*L. pneumophila*	Nursing home	SRR26709235	729,366	JAWVKG000000000	3,578,482	49	96	3,251	194,237	38.23	100	0.00
LP72_2016_4381	*L. pneumophila*	Nursing home	SRR26709234	632,025	JAWVKH000000000	3,368,858	34	79	3,045	500,349	38.26	100	0.58
LP73_2016_4407	*L. pneumophila*	Healthcare facility	SRR26709233	1,195,560	JAWVKI000000000	3,501,264	64	153	3,199	183,047	38.28	100	0.00
LP74_2016_4429	*L. pneumophila*	Healthcare facility	SRR26709232	632,704	JAWVKJ000000000	3,398,626	33	71	2,996	469,659	38.23	100	0.00
LP75_2016_7361	*L. pneumophila*	Ticket office	SRR26709230	#######	JAWVKK000000000	3,579,986	56	202	3,262	194,234	38.23	100	0.00
LP76_2016_7363	*L. pneumophila*	Ticket office	SRR26709229	865,283	JAWVKL000000000	3,578,173	44	115	3,250	194,234	38.24	100	0.00
LP77_2016_8723	*L. pneumophila*	Lodging facility	SRR26709228	636,238	JAWVKM000000000	3,565,442	87	88	3,223	86,441	38.3	100	0.00
LP78_2016_9232	*L. pneumophila*	Lodging facility	SRR26709227	589,218	JAWVKN000000000	3,535,373	46	79	3,199	237,642	38.24	100	0.00
LP79_2016_9249	*L. pneumophila*	Healthcare facility	SRR26709226	242,252	JAWVKO000000000	3,578,403	52	32	3,256	194,234	38.23	100	0.00
LP80_2016_9485	*L. pneumophila*	Healthcare facility	SRR26709225	266,405	JAWVKP000000000	3,477,957	44	38	3,123	214,484	38.32	100	0.58
LP81_2016_9487	*L. pneumophila*	Healthcare facility	SRR26709224	266,977	JAWVKQ000000000	3,570,770	30	29	3,223	299,313	38.3	100	0.58
LP83_2016_11187	*L. pneumophila*	Private residence	SRR26709223	212,089	JAWVKR000000000	3,531,035	80	30	3,184	79,659	38.3	100	0.00
LP84_2016_11195	*L. pneumophila* subsp. *fraseri*	Nursing home	SRR26709222	413,504	JAWVKS000000000	3,477,525	63	56	3,182	180,775	38.16	100	0.00
LP85_2016_11282	*L. pneumophila*	Ticket office	SRR26709221	417,248	JAWVKT000000000	3,578,176	47	57	3,255	194,234	38.23	100	0.00
LP86_2016_11,325A	*L. pneumophila*	Lodging facility	SRR26709219	435,165	JAWVKU000000000	3,485,254	36	59	3,136	300,987	38.32	100	0.00
LP88_2016_11484	*L. pneumophila*	Private residence	SRR26709218	478,296	JAWVKV000000000	3,357,037	34	56	3,027	336,533	38.3	100	0.00
LP89_2016_11555	*L. pneumophila*	Healthcare facility	SRR26709217	800,980	JAWVKW000000000	3,449,048	50	107	3,114	237,645	38.26	100	0.00
LP90_2016_12593	*L. pneumophila*	Private residence	SRR26709216	790,055	JAWVKX000000000	3,587,970	26	74	3,269	321,573	38.31	100	0.58
LP91_2016_12594	*L. pneumophila*	Private residence	SRR26709215	486,948	JAWVKY000000000	3,588,004	35	59	3,275	301,310	38.3	100	0.58
LP92_2016_12602	*L. pneumophila*	Private residence	SRR26709214	688,192	JAWVKZ000000000	3,588,618	31	71	3,269	321,573	38.3	100	0.58
LP93_2016_12604	*L. pneumophila*	Private residence	SRR26709213	479,542	JAWVLA000000000	3,555,874	33	60	3,237	336,534	38.26	100	0.00
LP94_2016_12605	*L. pneumophila*	Private residence	SRR26709212	520,029	JAWVLB000000000	3,588,474	30	65	3,270	501,617	38.3	100	0.58
LP95_2016_12642	*L. pneumophila*	Lodging facility	SRR26709211	430,693	JAWVLC000000000	3,485,412	37	53	3,139	300,987	38.32	100	0.00
LP96_2016_12645	*L. pneumophila*	Lodging facility	SRR26709182	494,430	JAWVLD000000000	3,485,913	42	55	3,146	258,962	38.32	100	0.00
LP97_2016_12647	*L. pneumophila*	Lodging facility	SRR26709180	303,560	JAWVLE000000000	3,312,012	49	38	2,962	189,901	38.28	100	0.19
LP98_2016_12649	*L. pneumophila*	Lodging facility	SRR26709179	963,576	JAWVLF000000000	3,483,354	26	126	3,131	300,987	38.33	100	0.00
LP99_2016_14554	*L. pneumophila*	County jail	SRR26709178	879,402	JAWVLG000000000	3,483,438	53	117	3,135	177,182	38.27	100	0.00
LP102_2016_17192B	*L. taurinensis*	Private residence	SRR26709177	2,154,655	JAWVLH000000000	3,228,131	37	194	3,004	281,117	47.89	99.97	0.78
LP103_2016_18,273A	*L. pneumophila*	Private residence	SRR26709176	625,855	JAWVLI000000000	3,344,388	25	89	3,024	638,047	38.24	100	0.58
LP105_2016_18,274A	*L. pneumophila*	Private residence	SRR26709175	6,017,450	JAWVLJ000000000	3,345,369	35	555	3,029	337,033	38.23	100	0.58
LP107_2017_92	*L. pneumophila*	Nursing home	SRR26709174	408,729	JAWVLK000000000	3,451,606	103	47	3,140	104,913	38.33	100	0.00
LP108_2017_94	*L. pneumophila*	Nursing home	SRR26709173	553,215	JAWVLL000000000	3,526,783	105	62	3,225	96,176	38.34	100	0.00
LP109_2017_96	*L. pneumophila*	Nursing home	SRR26709171	401,612	JAWVLM000000000	3,524,835	95	41	3,210	96,176	38.34	100	0.00
LP110_2017_100	*L. pneumophila*	Nursing home	SRR26709170	1,673,088	JAWVLN000000000	3,452,285	106	167	3,140	104,913	38.33	100	0.00
LP111_2017_2915_A1	*L. pneumophila*	Private residence	SRR26709266	1,274,472	JAWVLO000000000	3,499,307	48	180	3,185	218,744	38.28	100	0.00
LP112_2017_2921_A1	*L. pneumophila*	Private residence	SRR26709265	1,161,517	JAWVLP000000000	3,499,128	46	162	3,183	208,076	38.28	100	0.00
LP113_2017_3830	*L. pneumophila*	Lodging facility	SRR26709264	829,501	JAWVLQ000000000	3,432,783	18	97	3,085	510,411	38.21	100	0.58
LP114_2017_4027	*L. pneumophila*	Private residence	SRR26709263	622,732	JAWVLR000000000	3,465,835	44	72	3,145	244,094	38.28	100	0.00
LP116_2017_10304	*L. pneumophila* subsp. *fraseri*	Lodging facility	SRR26709262	616,945	JAWVLS000000000	3,474,144	37	56	3,157	212,670	38.16	100	0.00
LP118_2017_13076	*L. pneumophila*	Private residence	SRR26709261	641,676	JAWVLT000000000	3,468,034	37	77	3,139	301,913	38.29	100	0.00
LP119_2017_13083	*L. pneumophila*	Private residence	SRR26709260	510,330	JAWVLU000000000	3,467,767	45	57	3,146	257,885	38.29	100	0.00
LP121_2017_13917	*L. pneumophila*	Lodging facility	SRR26709259	896,224	JAWVLV000000000	3,467,702	45	82	3,143	242,848	38.29	100	0.00
LP122_2017_14194	*L. pneumophila*	Private residence	SRR26709258	552,311	JAWVLW000000000	3,514,193	66	49	3,186	137,469	38.26	100	0.00
LP123_2017_14198	*L. pneumophila*	Private residence	SRR26709257	421,041	JAWVLX000000000	3,509,320	29	53	3,166	319,975	38.25	100	0.00
LP124_2017_14204	*L. pneumophila*	Private residence	SRR26709255	492,851	JAWVLY000000000	3,331,398	46	64	2,977	196,896	38.24	100	0.00
LP125_2017_14205	*L. pneumophila*	Private residence	SRR26709254	827,115	JAWVLZ000000000	3,334,699	62	108	2,991	196,896	38.23	100	0.00
LP126_2017_14977	*L. pneumophila*	Private residence	SRR26709253	689,691	JAWVMA000000000	3,412,854	51	81	3,098	240,184	38.29	100	0.00
LP127_2017_14984	*L. pneumophila*	Lodging facility	SRR26709252	1,048,392	JAWVMB000000000	3,454,162	35	108	3,117	488,038	38.34	100	0.58
LP128_2017_15006	*L. pneumophila*	Lodging facility	SRR26709251	381,247	JAWVMC000000000	3,452,109	24	48	3,108	488,038	38.34	100	0.58
LP129_2017_15640	*L. pneumophila*	Private residence	SRR26709250	471,244	JAWVMD000000000	3,455,256	40	60	3,127	257,885	38.24	100	0.00
LP130_2017_15642	*L. pneumophila*	Private residence	SRR26709249	881,044	JAWVME000000000	3,455,955	33	121	3,121	380,366	38.24	100	0.00
LP132_2017_15856	*L. pneumophila*	Healthcare facility	SRR26709248	1,762,592	JAWVMF000000000	3,647,930	52	217	3,308	237,642	38.23	100	0.00
LP133_2017_15858	*L. pneumophila*	Healthcare facility	SRR26709247	383,066	JAWVMG000000000	3,410,745	119	48	3,099	81,249	38.31	100	0.00
LP134_2017_16867	*L. pneumophila*	Private residence	SRR26709246	758,068	JAWVMH000000000	3,529,729	53	105	3,205	213,677	38.23	100	0.00
LP137_2017_17239	*L. pneumophila*	Private residence	SRR26709244	1,305,904	JAWVMI000000000	3,633,347	74	174	3,319	193,077	38.28	100	0.00
LP138_2017_17845	*L. pneumophila*	Private residence	SRR26709243	378,881	JAWVMJ000000000	3,448,066	44	50	3,101	237,642	38.26	100	0.00
LP140_2017_19555	*L. pneumophila*	Private residence	SRR26709242	376,997	JAWVMK000000000	3,416,263	58	47	3,108	243,057	38.29	100	0.00
LP141_2017_19559	*L. pneumophila*	Private residence	SRR26709241	376,851	JAWVML000000000	3,415,366	60	48	3,109	165,077	38.28	100	0.00
LP143_2017_9736	*L. pneumophila*	Private residence	SRR26709240	500,228	JAWVMM000000000	3,530,252	66	51	3,214	183,098	38.23	100	0.00
LP144_2017_20670	*L. pneumophila*	Private residence	SRR26709239	1,380,338	JAWVMN000000000	3,375,376	33	120	3,047	336,780	38.27	100	0.00
LP149_2006_8538	*L. pneumophila*	Healthcare facility	SRR26709210	450,584	JAWVMO000000000	3,456,343	71	57	3,136	155,258	38.31	100	0.58

^*a*^ Determined using PGAP.

^*b*^ Determined using bbmap (17).

^*c*^ Determined using Quast.

^*d*^ Determined using CheckM.

## Data Availability

Sequences were publicly deposited in the NCBI database with BioProject accession number PRJNA1036263. For each strain, Table 1 reports the GenBank and SRA accession numbers. For privacy reasons, more detailed information on the isolation position of the different strains may be provided following evaluation of the request by the corresponding author.
